# HIV-TB the deadly duo, the biggest health challenge in Fiji

**DOI:** 10.1186/1471-2334-12-S1-O22

**Published:** 2012-05-04

**Authors:** Santhakumari Muller, Vinod Narayan Sami

**Affiliations:** 1 Department of Health Sciences, College of Medicine, Nursing and Health Sciences, Fiji National University, Suva, Fiji

## Background

Tuberculosis is a common opportunistic infection and HIV patients with latent TB infection are at risk of reactivation and those with recently acquired infection are at high risk of progressive primary TB. Approximately 1000 people in the western pacific region die from the disease every day. The threat of increasing HIV rates fuelling the TB epidemic has become an important massive challenge in Fiji to the control of TB at all levels. Hence this study was to evaluate the rate of TB co infection among HIV patients in Fiji.

## Methods

This study involved the retrospective descriptive analysis of the data available in the PJ Twomey Hospital, Suva, Fiji. All registered cases of HIV and HIV-TB has been included in the study.

## Results

By 2010, Fiji has 191 registered cases of tuberculosis and 393 HIV positive. The % of TB patients with known HIV status is 100 because of the recent Fiji HIV/TB surveillance policy which recommends that HIV testing is mandatory in all newly diagnosed cases of TB for all health care settings. Fiji has reported 2% of new TB patients every year which are also HIV positive and the mortality rate of HIV/TB patients is very high (80%).

## Conclusion

The HIV positive co-infected with TB have developed active TB than patients who are HIV negative. Hence the TB/HIV co-infected were started TB therapy prior to ARVs. TB treatment reduces the burden of HIV in people living with TB.

